# Case reports on a gastric mass presenting with regional lymphadenopathy: A differential diagnosis is gastric schwannoma

**DOI:** 10.1016/j.ijscr.2020.05.098

**Published:** 2020-06-12

**Authors:** Arnav Wadhawan, Maureen Brady, Charles LeVea, Steven Hochwald, Moshim Kukar

**Affiliations:** Roswell Park Comprehensive Cancer Center, Buffalo, NY, USA

**Keywords:** Schwannoma, Gastrointestinal schwannoma, Neurinoma, Neurilemmoma, Gastrointestinal stromal tumor, Gastric mass with regional lymphadenopathy

## Abstract

**Background:**

Gastric schwannomas are uncommon among a broad range of possible diagnoses in the work up of a gastric mass. Regional lymphadenopathy associated with gastric schwannoma is an even less common occurrence and one would otherwise suspect a malignant neoplasm.

**Case presentation:**

We present two non-consecutive cases from a signle academic center depicting Caucasian females in their 5th and 6th decades of life with gastric schwannoma and adjacent lymphadenopathy. Multiple lymph node excisions were performed without evidence of neoplasia.

**Discussion:**

Lymphadanopathy in the presence of a gastric mass typically represents malignant neoplasm. A less than likely presentation of gastric schwannoma with reactive regional lymph nodes poses a challenge to adequate preoperative diagnosis and increases risk for a more aggressive than necessary surgical approach with lymphadenectomy.

**Conclusion:**

While the correlation between gastric schwannoma and lymphadenopathy is uncertain, this ought to be considered. If diagnosis can be confirmed preoperatively, omission of lymphadenectomy is appropriate.

## Highlights

• Gastric schwannoma with regional lymphadenopathy is an uncommon presentation.
• Excision of peri gastric lymph nodes confirmed reactive, nonneoplastic etiology.
• Correlation between gastric schwannoma and enlarged lymph nodes remains unclear.
• Omission of lymphadenectomy is less aggressive and curative in gastric schwannoma.

## 1 Introduction

Schwannomas, also known as neurinomas and neurilemmomas, are the most common benign solitary peripheral nerve tumors that can originate from any nerve with a Schwann cell sheath [1]. It is typical for schwannomas to occur in the head and neck most frequently originating from peripheral nerves and connective tissue [2]. A subset of schwannomas can arise in the stomach but tend to be a rare occurrence [2]. The conventional diagnosis of a gastric schwannoma is made incidentally on investigations for upper gastrointestinal complaints. A far less common presentation is an asymptomatic gastric mass with regional lymphadenopathy which poses a diagnostic and therapeutic dilemma [1,2]. Here, we detail management of two patients representing the latter clinical scenario and review of the literature in detail. This work has been reported in line with the SCARE criteria [3].

## 2 Case presentation

### 2.1 Case 1

**Fig. 1:**
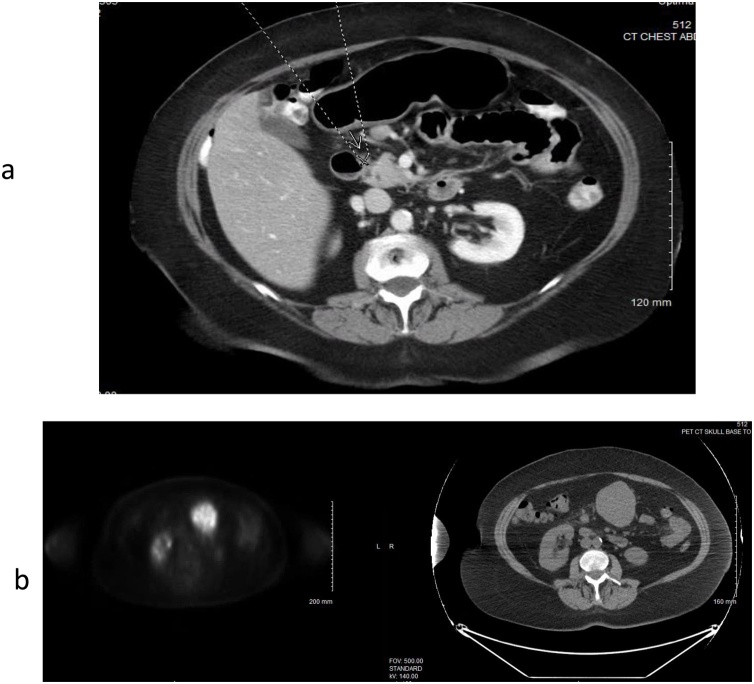
(a) Computerized Tomography section showing enlarged perigastric lymph nodes. (b) PET scan showing intense uptake in primary gastric lesion.

A 66 year old Caucasian female was referred to our institute for an incidental 5 cm gastric antral mass identified on an elective laparoscopic sleeve gastrectomy for weight management; elective surgery was aborted. Intraoperative findings were also remarkable for multiple diminutive white nodules on the surface of the stomach, small intestine, and peritoneal wall of the left upper quadrant. Biopsies returned mesothelial hyperplasia. She shared complaints of intermittent but chronic nausea and constipation. Physical exam was unremarkable. Prior surgical history consisted of removal of an ovarian cyst. Medical, social, and family histories were noncontributory. Esophagogastroduodenoscopy (EGD) identified a subepithelial bulge within the gastric antrum along the greater curvature of the stomach 5 cm proximal to the pylorus. On endoscopic ultrasound (EUS), the mass appeared heterogeneous arising from the muscularis propria layer of the stomach. Fine needle aspiration (FNA) revealed atypical spindle cells in a hypocellular specimen most consistent with a spindle cell lesion. Immunostains CD117, C-KIT, CD 34, smooth muscle actin, and S100-DAB were non-contributory due to scant cellularity. Of note, there were two hypoechoic perigastric lymph nodes at the level of the gastric antrum 14 mm and 6 mm in size. EUS guided trans-gastric FNA of the dominant lymph node was negative for malignancy. Contrast enhanced computed tomography (CT) scan of the chest, abdomen, and pelvis demonstrated an exophytic gastric body mass with adjacent mild mesenteric adenopathy, sub- and retro pyloric and gastroduodenal distributions (Fig. 1a). Subsequent 18- fluorodeoxyglucose (FDG) positron emission tomography (PET) scan indicated intense abnormal FDG uptake (SUVmax 11.7) within the gastric mass. No definitive abnormal FDG uptake within adjacent mesenteric adenopathy (Fig. 1b).

**Fig. 2:**
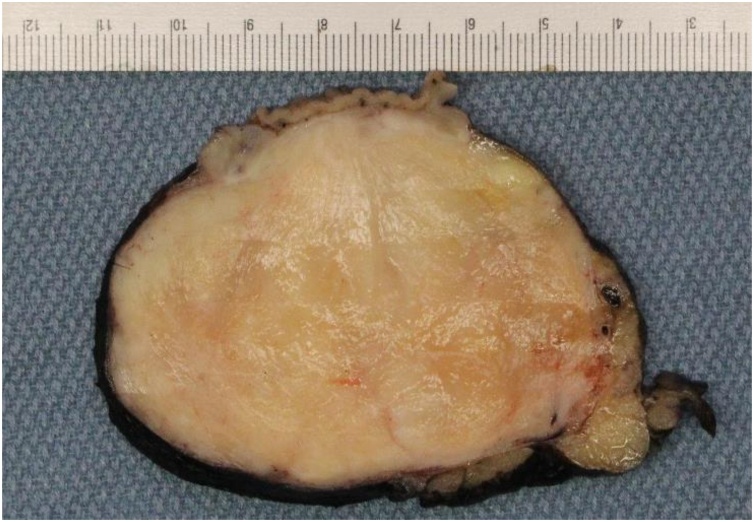
Gross appearance of the schwannoma on cut section.

**Fig. 3:**
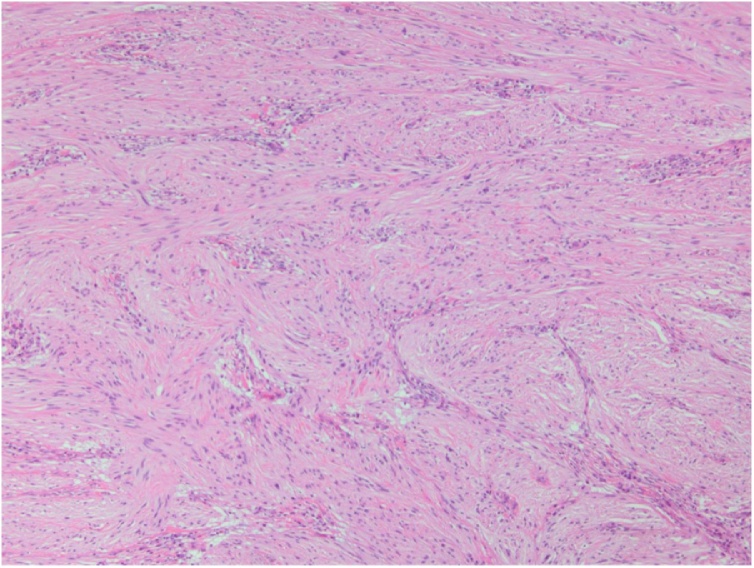
H&E appearance of the tumor. Photomicrographs of the microscopic appearance of the tumors were taken at 100X magnification.

With a working diagnosis of gastrointestinal stromal tumor (GIST), she was offered partial gastrectomy for definitive diagnosis and therapeutic intent in conjunction with sleeve gastrectomy by the patient’s outside surgeon considering that it was feasible and per her initial wishes. Intraoperatively, thickened and chronically inflamed lymph nodes were appreciated along the lateral aspect of the mass. Multiple lymph node specimens were sent for frozen section and returned negative. Grossly, the well-circumscribed firm gastric subserosal nodule measured 8.2 × 6.8 × 4.7 cm with unremarkable appearing overlying mucosa. Cut surface exhibited a white-tan appearance (Fig. 2). Microscopic evaluation revealed a schwannoma with two mitoses per fifty high power fields. A total of four perigastric lymph nodes were excised without abnormality. Immunohistochemical (IHC) staining revealed tumor cells were positive for S100-DAB and negative for C-KIT. Histopathological and immunohistochemical features were consistent with the presence of a gastric schwannoma with reactive lymphadenopathy (Fig. 3). The patient made a full recovery uneventfully; Clavien-Dindo score of 1.

### 2.2 Case 2

**Fig. 4:**
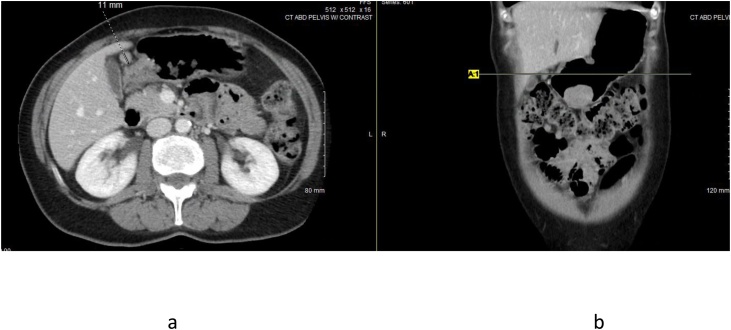
Computerized tomography image showing enlarged perigastric lymph node (a) and primary tumor (b).

**Fig. 5:**
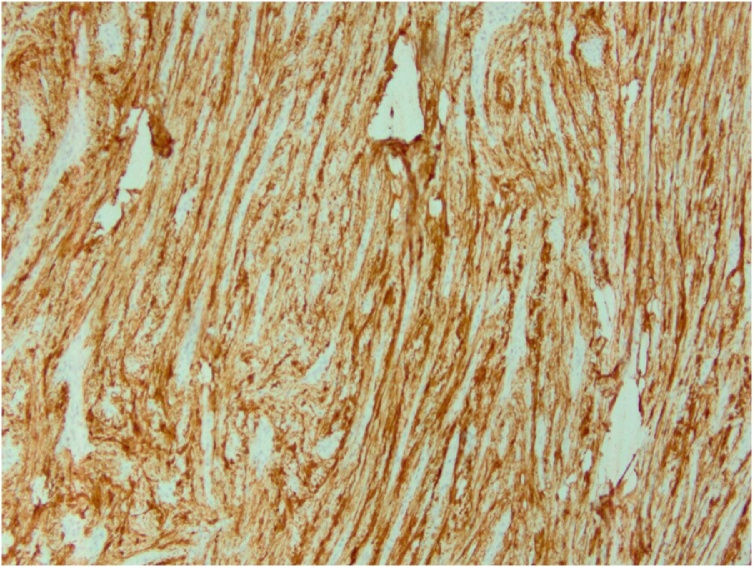
Tumor positively staining for S100.

59 year old Caucasian female presented with melena and acute anemia. Initial EGD revealed a 3 cm mass and overlying erosions at the junction of the gastric antrum and body along the greater curve. FNA was nondiagnostic. She received a blood transfusion and stabilized on oral iron supplements. She was otherwise asymptomatic with normal physical findings. Medical, surgical, family and social histories were noncontributory. A repeat EGD with EUS was performed which described a round subepithelial antral mass. Sonographically, a 35 × 33 mm hypoechoic and irregular lesion appeared to originate from the muscularis propria. Note, two minimally enlarged perigastric lymph nodes were visualized; the largest measured 7 × 5 mm. FNA of the gastric mass was negative for carcinoma with presence of lymphoid tissue. IHC stains were negative for neuroendocrine tumor, GIST, and leiomyoma. Subsequent contrast enhanced CT of the abdomen and pelvis revealed a homogeneously enhancing rounded predominantly intraluminal mass involving the anterior gastric wall measuring 3.6 × 4.1 cm. Several perigastric lymph nodes were noted measuring up to 1.1 cm (Fig. 4a and b). Ultrasound guided core needle biopsy of the gastric mass was consistent with schwannoma, S100 positive. In the setting of a gastric mass resulting in gastrointestinal bleed and clinical concern for regional adenopathy representing lymphoproliferative disease versus reactive change, she underwent a therapeutic laparoscopic partial gastrectomy with excision of adjacent lymph nodes for definitive diagnosis. Gross examination of the gastric specimen revealed a well-circumscribed homogeneous pink-tan nodule, 4.1 × 3.7 × 3.1 cm. Microscopic and immunohistochemical features coincided with results of core biopsy (Fig. 5). A total of six perigastric lymph nodes excised were negative for neoplasia. Postoperative course was uncomplicated with a complete recovery; Clavien-Dindo score of 1.

## 3 Discussion

Many different types of tumors can be found in the stomach such as epithelial tumors, lymphomas and a wide range of mesenchymal tumors. Gastric mesenchymal tumors can be divided among four categories: the true smooth muscle tumors (leiomyomas, glomus tumors, leiomyosarcomas), neurogenic tumors (schwannomas, neurofibromas, ganglioneuromas, paragangliomas), fibroblastic tumors (desmoid, inflammatory myofibroblast tumors), and gastrointestinal stromal tumors [4]. GISTs and gastric schwannomas share a predilection for the stomach which can create confusion in differentiating the two [5]. This can lead to misdiagnosis of gastric schwannomas as GISTs as they lack certain morphological features characteristic of the schwannomas seen elsewhere in the body [6]. Additional qualities shared by gastric schwannomas and GISTs contribute to a diagnostic quandary: predominant occurrence in patients aged 40–60 years, lack of distinct clinical features, and appearance on CT [2,7].

Gastric Schwannomas are neurogenic tumors and are thought to arise from the sheath of Auerbach’s plexus or the sheath of Meissner’s plexus [1,2]. The incidence rate of gastric schwannomas is approximately 0.2% of all gastric tumors and majority present as benign, slow growing and asymptomatic tumors [1,8]. Gastric schwannomas test positive for the S-100 protein and negative for CD117, CD34, Desmin, and smooth muscle actin [9,10].

Gastric schwannomas are generally benign and have excellent prognosis after resection [5]. A diagnostic and therapeutic dilemma exists when gastric masses present with regional lymphadenopathy. This is commonly indicative of a malignant neoplasm unless proven otherwise.

However, evident in the reports above and limited data available in the literature, adenopathy is invariably reactive in this setting. The correlation between gastric schwannoma and enlarged lymph nodes remains unclear. Hou Y et al. report on gastrointestinal schwannomas shows that 97% of these tumors present with peripheral cuff like lymphoid aggregates. Lymphocytes are aggregated around the periphery of the tumor and form the characteristic feature of a lymphoid cuff which is not seen in soft tissue and central nervous system schwannomas. The reason for this infiltration is not clear. Most speculate that the lymphoid cuff might result from cytokines secreted by the tumor cells which induce chemokinesis of lymphocytes [11,12].

Since this is reactive adenopathy, an aggressive surgical approach with regional lymphadenectomy is not warranted. A thorough preoperative workup including an endoscopic ultrasound guided fine needle aspiration is critical to confirm the diagnosis and a wedge resection of stomach, if feasible, should suffice as a definitive treatment. In the literature there are some reports of formal gastrectomy and regional lymphadenectomy which seems like an aggressive management option for a benign tumor with no case reports in literature showing tumor involvement of lymph nodes [13,14]. If a confident preoperative diagnosis cannot be made, as demonstrated in patient 1, intraoperative evaluation of lymph nodes by frozen section is helpful in choosing the surgical extent of the operation.

## 4 Conclusion

In conclusion, gastric schwannoma should be considered in the differential diagnosis of gastric masses presenting with regional lymphadenopathy. EUS-FNA can establish the diagnosis in most cases and if confirmed, a less aggressive surgical approach including omission of lymphadenectomy serves curative in these patients.

## Declaration of Competing Interest

None.

## Funding

None.

## Ethical approval

The study is exempt from ethical approval in our institution.

## Consent

Written informed consent was obtained from the patients for publication of this case report and accompanying images. A copy of the written consent is available for review by the Editor-in-Chief of this journal on request.

## Author contribution

Arnav Wadhawan: Resources, acquisition of data, writing - original draft, final approval of the version to be submitted. Maureen Brady: Resources, acquisition of data, writing - original draft, writing - review and editing, final approval of the version to be submitted. Charles LeVea: Acquisition of data, analysis and interpretation of data, final approval of the version to be submitted. Steven Hochwald: Acquisition of data, analysis and interpretation of data, final approval of the version to be submitted. Moshim Kukar: Project administration, acquisition of data supervision, conceptualization, analysis and interpretation of data, writing original draft, final approval of the version to be submitted.

## Registration of research studies

This was not required in previous submissions.

## Guarantor

Moshim Kukar.

## Provenance and peer review

Editorially reviewed, not externally peer-reviewed.
